# Problems of creating antibody phage libraries and their solutions

**DOI:** 10.18699/vjgb-24-29

**Published:** 2024-04

**Authors:** V.S. Aripov, N.V. Volkova, A.A. Ilyichev, D.N. Shcherbakov

**Affiliations:** State Research Center of Virology and Biotechnology “Vector”, Koltsovo, Novosibirsk Region, Russia; State Research Center of Virology and Biotechnology “Vector”, Koltsovo, Novosibirsk Region, Russia; State Research Center of Virology and Biotechnology “Vector”, Koltsovo, Novosibirsk Region, Russia; State Research Center of Virology and Biotechnology “Vector”, Koltsovo, Novosibirsk Region, Russia

**Keywords:** phage library, bacteriophage replicative form, monoclonal antibodies, helper phage, competent cells, фаговая библиотека, репликативная форма бактериофага, моноклональные антитела, фаг-помощник, компетентные клетки

## Abstract

Phage display has become an efficient, reliable and popular molecular technique for generating libraries encompassing millions or even billions of clones of divergent peptides or proteins. The method is based on the correspondence between phage genotype and phenotype, which ensures the presentation of recombinant proteins of known amino acid composition on the surface of phage particles. The use of affinity selection allows one to choose variants with affinity for different targets from phage libraries. The implementation of the antibody phage display technique has revolutionized the field of clinical immunology, both for developing tools to diagnose infectious diseases and for producing therapeutic agents. It has also become the basis for efficient and relatively inexpensive methods for studying protein–protein interactions, receptor binding sites, as well as epitope and mimotope identification. The antibody phage display technique involves a number of steps, and the final result depends on their successful implementation. The diversity, whether natural or obtained by combinatorial chemistry, is the basis of any library. The choice of molecular techniques is critical to ensure that this diversity is maintained during the phage library preparation step and during the transformation of E. coli cells. After a helper phage is added to the suspension of transformed E. coli cells, a bacteriophage library is formed, which is a working tool for performing the affinity selection procedure and searching for individual molecules. Despite the apparent simplicity of generating phage antibody libraries, a number of subtleties need to be taken into account. First, there are the features of phage vector preparation. Currently, a large number of phagemid vectors have been developed, and their selection is also of great importance. The key step is preparing competent E. coli cells and the technology of their transformation. The choice of a helper phage and the method used to generate it is also important. This article discusses the key challenges faced by researchers in constructing phage antibody libraries

## Abstract (ru)

Фаговый дисплей стал эффективной, надежной и востребованной молекулярной техникой для создания библиотек, содержащих миллионы или даже миллиарды клонов, экспонирующих различающиеся пептиды или белки. В основе этого метода лежит соответствие между генотипом и фенотипом фага, обеспечивающее презентацию на поверхности фаговых частиц рекомбинантных белков с известным аминокислотным составом. Использование процедуры аффинной селекции позволяет проводить отбор из фаговых библиотек вариантов, обладающих сродством к различным мишеням. Внедрение технологии фагового дисплея антител имело революционное значение в области клинической иммунологии, как для создания инструментов диагностики инфекционных заболеваний, так и для получения терапевтических агентов. Она стала также основой эффективных и относительно недорогих методов исследования белок-белковых взаимодействий, сайтов связывания рецепторов, идентификации эпитопов и мимотопов. Технология фагового дисплея антител включает в себя ряд этапов, от успешной реализации которых зависит финальный результат. Основа любой библиотеки – разнообразие, природное или полученное при помощи методов комбинаторной химии. Критически важным является подбор молекулярных техник, обеспечивающих сохранение этого разнообразия на этапе получения библиотек фагмид и на этапе трансформации клеток E. coli. После добавления фага-помощника к суспензии трансформированных клеток E. coli происходит формирование библиотеки бактериофагов, которая служит рабочим инструментом для проведения процедуры аффинной селекции и поиска индивидуальных молекул. Несмотря на кажущуюся простоту создания фаговых библиотек антител, существует ряд тонкостей, которые необходимо учитывать. В первую очередь это особенности подготовки фагмидного вектора. В настоящее время разработано большое количество фагмидных векторов, и их выбор также имеет большое значение. Ключевым этапом считается подготовка компетентных клеток E. coli и технология их трансформации. Немаловажен выбор фага-помощника и способа его подготовки. Статья посвящена основным проблемам, с которыми сталкиваются исследователи при создании фаговых библиотек антител.

## Introduction

In 1985, Smith was the first to describe the phage display technique and show that filamentous phages are able to display a peptide of interest on their surface after insertion of a foreign DNA fragment into the major coat protein gene (Smith, 1985). Subsequently, Parmley and Smith described the process for selecting and enriching phage libraries based on affinity binding called “biopanning” (Parmley, Smith, 1988). And in 1991, McCafferty and Winter were the first to use phage display technology for antibody production, having generated their combinatorial libraries using filamentous bacteriophages to screen for antigen-specific monoclonal antibodies (McCafferty et al., 1990).

The filamentous bacteriophages M13, f1, and fd were key tools of phage display technology. They are stable in solutions under various conditions, including extreme pH and high temperatures (Brigati, Petrenko, 2005), and insensitive to lytic enzymes (including DNases and proteases) (Larocca et al., 2002).

**Fig. 1.**
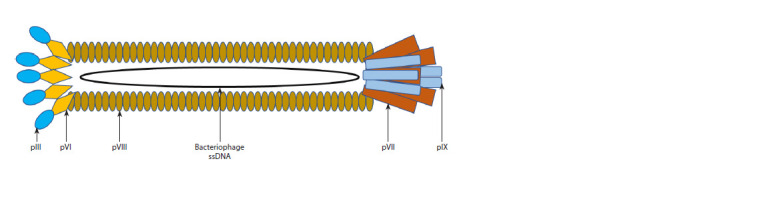
Schematic representation of the filamentous bacteriophage M13. The filamentous phage M13 is approximately 880 nm long and 6.5 nm wide and is surrounded by single-stranded DNA. The capsid of bacteriophage M13 is formed by five copies of different minor coat proteins (pIII, pVI, pVII, and pIX), but the major coat protein pVIII, represented by ~2,700 copies, makes up the most part of the capsid (Chung et al., 2011).

The filamentous bacteriophage M13 (Fig. 1) is one of the most commonly used bacteriophages for antibody phage display (Sokullu et al., 2019). M13 phage has a high replication capacity, and large foreign DNA fragments can be inserted into its genome by genetic engineering methods. This bacteriophage is non-lytic, infects and replicates in Escherichia coli strains carrying F+ that form F-pili (O’Callaghan et al., 1973). The greatest portion of the virion consists of singlestranded DNA covered with approximately 2,700 copies of the major coat protein pVIII. Each end of the phage consists of five copies of two different proteins: pVII and pIX at one end, pIII and pVI at the other end (see Fig. 1). Virion length depends on the length of the genome packed into a phage particle. Up to 12,000 nucleotides can be added to the wildtype genome without disrupting phage packaging (Rasched, Oberer, 1986).

During the replication cycle of filamentous bacteriophages, their genome is simultaneously present in the bacterial cell as a large number of copies of the replicative form (doublestranded circular DNA) and single-stranded DNA (ssDNA). The presence of ssDNA in the DNA sample interferes with cloning as it creates a “background” of clones without insertion of the target gene. Using the purified replicative form, it is possible to clone DNA fragments within the sites encoding the bacteriophage protein of interest, mainly the pIII and pVIII proteins. Such vector systems are referred to as systems 3 and 8, respectively (Kay et al., 1996).

The main differences in using the pIII and pVIII proteins consist in the length of foreign peptides, presentation of chimeric proteins on the surface of the phage progeny, and the effect on viability (Ilyichev et al., 1989; Bass et al., 1990; Barbas et al., 1991). Compared to the pVIII protein, the pIII protein can ensure representation of longer amino acid sequences (Näkelä et al., 1978). The pVIII protein system is commonly used for small peptides. Larger polypeptides interfere with the function of the pVIII protein, so a system with the pIII protein is used for them. Due to the structural properties of Ff phages, application of the pIII protein in the phage display system results in less than five copies of the fusion protein being present in the phage progeny. It is only in the absence of the pIII gene in the helper phage genome that five copies of this protein will be present in the phage progeny (Smith, 1993).

The use of pVIII allows production of phage particles containing much more than five copies of chimeric proteins: hundreds or even thousands of them (Veronese et al., 1994). However, because of the multiple copies of chimeric proteins, the avidity effect begins to play a major role, which can impede the selection of protein variants with different affinities. Therefore, pVIII is used in phage display to expand the range of potential ligands, and pIII is used to reduce or eliminate the avidity effect to select high-affinity proteins. In addition, the N-terminal domain of the major coat protein pIII is involved in host cell infection. To infect E. coli, filamentous phages use filamentous bacterial structures known as F-pili. The end of the phage with the pIII protein interacts with the TolA protein on the bacterial surface. Although the pIII protein is relatively large, excessive perturbation of its structure can lead to disruption of its interaction with F-pili. This can have a significant effect on progeny phage viability; therefore, applications of the pIII protein for protein fragment display are limited. Such problems do not occur when the VIII protein is used.

As for the application of other bacteriophage proteins, very few papers have so far reported the feasibility of using the pVII and pIX proteins (Gao et al., 1999, 2002). The pVII and pIX proteins form a complex at the end of the phage particle opposite to the end carrying the widely used pIII protein. In order to demonstrate the feasibility of using these proteins for display, a phagemid variant was developed in which the variable regions of the heavy and light chains of the antibody were fused to the N-termini of pVII and pIX, respectively. Remarkably, the fusion proteins interact to form a functional Fv-binding domain on the phage surface. This approach looks promising for displaying complex libraries of peptides and proteins that could form combinatorial heterodimeric structures of the Fv domain of an antibody. However, this format has not yet been widely used.

**Fig. 2.**
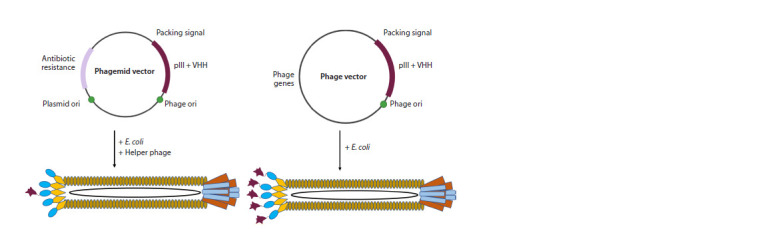
Distinguishing between a phagemid and a phage vector.

In addition to phage vectors, phagemids are also used for library construction. Phagemids are vector molecules combining the properties of a plasmid and a phage vector (Kay et al., 1996). The main difference between phagemids and phage vectors is that phagemids are vectors derived from Ff-phages that contain the plasmid origin of replication (Ori) for doublestranded replication and the f1 Ori to allow single-stranded replication and packaging into phage particles (Fig. 2). They usually either do not encode at all, or encode only one type of major coat protein, and other structural and functional proteins required to complete the bacteriophage life cycle are provided by the helper phage (Ledsgaard et al., 2018).

An alternative approach is proposed by Chasteen and his team, who have developed “bacterial packaging cell lines” containing M13-based helper plasmids (Chasteen et al., 2006). The use of such cells ensures the synthesis of phage packaging proteins that assemble phage particles as efficiently as a helper phage infection, thereby eliminating the helper phage stage of bacterial cell infection.

Other elements, such as molecular tags and selective markers, are also inserted into the phagemids to facilitate subsequent operations, such as protein purification. Tagging can also be used to facilitate library screening

The relatively small size of phagemids allows one to clone larger gene fragments encoding fusion proteins. The efficiency of phagemid transformation is higher than that of phage vectors, allowing the construction of large repertoire libraries (Qi et al., 2012).

There are a large number of phagemid variants. Type III and VIII phagemids can be reviewed in more detail in (Qi et al., 2012). The most common phagemids are pHEN, pComb, pSEX, and pADL. Each of them has several derivatives. Each phagemid has an original design, the choice of which depends on the researcher’s goal. Thus, the pADL-10b phagemid has f1 ori to enable single-stranded replication and packaging into phage particles, a copy of LacI to provide lac promoter repression, and a strong transcription terminator to prevent unwanted and toxic expression during cloning. This design ensures reliable cloning of variant antibody genes and increased library stability during screening (Krebber et al., 1997). This vector is recommended for scFv display and is best suited when a re-cloning step is envisaged to synthesize the protein in a soluble form.

Incorporation of the amber stop codon (TAG) between the target protein and the phage coat protein initiates expression of the fusion protein in E. coli suppressor strains such as TG1. In this case, the TAG codon is translated as a glutamine residue. However, in non-suppressor strains, such as HB2151, a soluble form of the recombinant protein will be produced because such strains do not contain glutamine in the amber stop codon. In this case, TAG would be the normal stop codon (Hoogenboom et al., 1991).

The expression level of the pADL series phagemids is equivalent to that of the pComb3 phagemid and significantly lower than that of the pHEN phagemid. Overexpression leads to toxicity, which makes antibody libraries genetically unstable and contributes to a strong tendency towards loss of diversity. In this respect, the pADL series phagemids provide a good balance between toxicity and synthesis of target antibodies in the periplasmic space of bacterial strains (Ishina et al., 2020).

The most popular derivative of the pComb vector is pComb3XSS. It contains two hydrolysis sites of the SfiI restrictase, which recognizes the GGCCNNNNNGGCC sequence. The lack of specificity for the five central nucleotide pairs allows PCR products encoding antibody fragments to be inserted in the desired orientation. The orientation is determined by the design of the forward and reverse primers, which also contain the SfiI site. The Comb3XSS vector also contains 6×His and HA tags, which allow protein purification and detection. It also contains an amber stop codon to switch off expression of the pIII fusion protein by switching to a non-suppressor E. coli strain, allowing production of soluble protein without subcloning.

The key points in working with phage libraries are vector preparation, preparation of well-competent cells, electroporation and amplification of the helper phage. Each of these points is discussed below and some recommendations are provided.

## Phagemid preparation

Preparing a vector for insertion of target DNA sequences is one of the key aspects in phage library construction. The main difficulty consists in purifying the phagemid DNA preparation from single-stranded and linear forms of DNA. These DNA forms constitute the “background” on the control dish during electroporation, making it difficult to assess the “representation” of the phage library. Most importantly, they can have a negative impact on the level of library diversity. Various phage DNA purification protocols are used to overcome this problem. Once DNA has been isolated, it is possible to purify the preparation from the single-stranded and linear form using the phenol method. This is the “classic” method, but it has a number of disadvantages, such as significant losses of the replicative form of DNA, and handling phenol requires special working conditions. DNA isolation using the phenol method often depends on the skill of the person performing this procedure (Maniatis et al., 1984).

There is also a method of isolating the replicative form of phage DNA using ammonium acetate. Unlike the phenol method, it does not require special working conditions but is rather time-consuming (Maniatis et al., 1984).

**Fig. 3.**
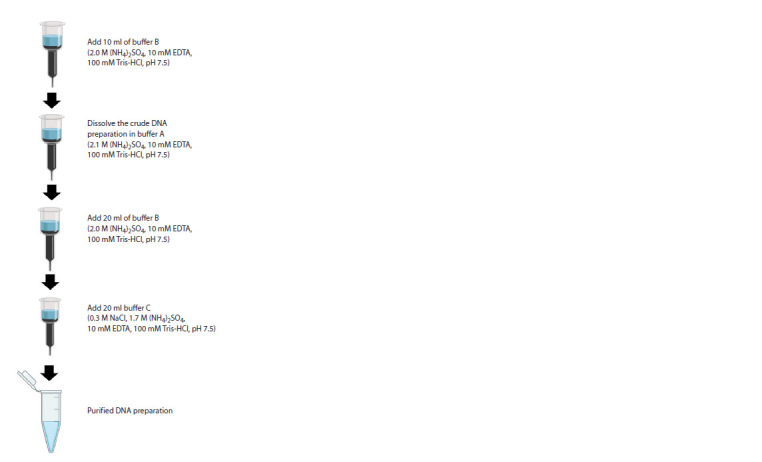
Scheme of phage vector purification to remove single-stranded and linear forms of DNA.

An alternative is to purify the DNA preparation using sorbents (e. g., HiTrap PlasmidSelect Xtra Sorbent). The main steps of DNA purification using a sorbent are shown in Figure 3.

This method is not ideal either, but it does not require any special conditions to carry out the purification procedure. After purification on the sorbent, one should be prepared for significant DNA loss. In the best-case scenario, the total DNA yield will be 10 %. Meanwhile, this amount of DNA purified from satellite forms is quite sufficient to obtain a phage library.

## Competent cells

Highly competent cells are a critical step in obtaining a phage library with high representation. TG1 cells are considered to be among the best cell lines for obtaining large phage libraries (Tu et al., 2005; Chai et al., 2020). TG1 is an unmodified derivative of the E. coli strain JM101. Currently, TG1 is one of the fastest growing E. coli clones, which undoubtedly plays an important role in setting up the experiment

**Fig. 4.**
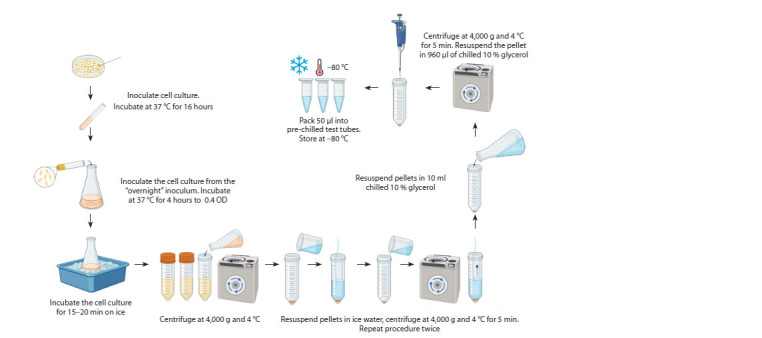
Typical scheme for obtaining competent cells.

There are commercial companies offering ready-made cells with a competence of ≥4·1010 CFU/μg DNA. However, the supply of such cells is not always possible, and the transport process leads to temperature fluctuations that reduce cellular competence. Therefore, the development of a protocol for obtaining highly competent cells for the generation of phage antibody libraries is an extremely important task (Fig. 4).

To obtain a large diversity of the phage library (1010– 11 CFU), it is necessary to observe simple but very important conditions in the preparation of competent cells. It is important to ensure proper culturing conditions, and temperature in particular. The recommended temperature for culturing is +37 °C. It is also important to ensure that the cell culture density does not exceed 0.4 OD. Cells should be in the early to mid logarithmic phase of growth. Using cells in the late logarithmic or stationary phase will significantly reduce electroporation efficiency. It is also very important to avoid temperature extremes. Once your culture has grown to the required density, all manipulations need to be carried out on “wet” ice.

It is also necessary to resuspend the cells very carefully at each stage, since they are very fragile and easily destroyed. It is recommended to store the obtained cells at –80 °C (Chai et al., 2020). At this temperature, the cells retain their competence for a longer time. However, cellular competence deteriorates over time, so freshly prepared cells should be used to maintain the diversity of phage libraries.

## Electroporation

**Fig. 5.**
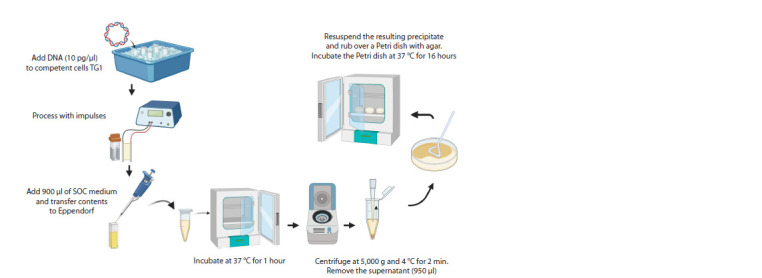
Scheme of electroporation

Prior to electroporation (Fig. 5), its efficiency was assessed using pUC19 plasmid DNA at a known concentration when a phage library was obtained (Chai et al., 2020).

Before starting electroporation, it is necessary to thaw the competent cells in ice. This manipulation preserves the viability of the cells. Simultaneously with thawing the cells, any nutrient medium needs to be warmed to +37 °C.

During electroporation, it is important to carry out the manipulations clearly and quickly. First, 1 μl (10 pg/μl) of DNA needs to be added. This is the amount needed to deliver the control plasmid pUC19 to the thawed electrocompetent cells. If electroporation is performed with a ligase mixture that has been pre-cleared from the ligation buffer, it is necessary to take much larger (15–25-fold) amounts of DNA (Pardon et al., 2014).

It is also important to control the voltage according to the cuvette size. Immediately after the electrical pulse, 1 ml of SOC recovery medium must be added and the cells need to be incubated for 1 hour at +37 °C in a thermostat without stirring. After incubation, the transformed cells are centrifuged, 950 μl of supernatant is removed, the precipitate is resuspended and spread on a Petri dish containing agar, the required antibiotic and 2 % glucose. Incubation is performed at +37 °C for 16 hours

On the following day, the electroporation efficiency is assessed. Electroporation efficiency is defined as the number of colony-forming units obtained by electroporation of 1 μg of plasmid per given volume of competent cells. The higher the competence of the cells obtained, the greater the diversity of the phage library. Cells above 1010 CFU are considered highly competent

## Helper phage

Together with the phagemid, the helper phage is an important component for successful production of diverse libraries. It is required for the assembly of phage particles by infecting cells with phagemid. The helper phage carries all the genes required for infection, replication, assembly and budding, thus providing the phagemid, which carries the gene encoding the pIII-scFv fusion protein, with the proteins required for amplification.

The helper phage infects the bacterium by first attaching to its F-pili and then, once attached, transporting its genome into the cytoplasm of the host cell. Inside the cell, the phage genome initiates the production of phagemid single-stranded DNA in the cytoplasm. The phagemid DNA is then packaged into phage particles. Phage particles containing singlestranded DNA are released from the bacterial cell into the extracellular environment. Filamentous phages inhibit bacterial growth but, unlike phage λ and phage T7, do not lyse host cells. Helper phages are usually designed so that their DNA packaging is less efficient than that of phagemids, and the resulting phage particles preferentially contain phagemid DNA. This is due to a defective replication start site (Lund et al., 2010).

M13KO7 is the main helper phage currently used in laboratories (Russel et al., 1986; Vieira, Messing, 1987; Du, Zhang, 2009). All other helper phages are derived from it. The M13KO7 helper phage is a derivative of phage M13 and carries a heterologous, low copy number p15A replication start site. When the M13KO7 phage is present alone in the host bacterium, replication is sufficient to produce its high titers. However, if a large number of copies of the phagemid are present, it will displace the helper phage genome during packaging, meaning that most of the resulting phage particles will carry phagemid DNA (Vieira, Messing, 1987). The capsid of the resulting phage particles will contain pIII encoded by the helper phage genome as well as the chimeric pIII-scFv protein encoded by the phagemid. In practice, there is preferential incorporation of wild-type pIII from the helper phage. This means that most phage particles will be “bald” (lacking the chimeric pIII-scFv). The preferred display of pIII over fused pIII-scFv is probably due to a combination of differences in expression/translation levels and the fact that some of the fused pIII-scFv appear to be degraded.

Choosing a specific helper phage to work with is quite difficult. Despite the stated differences in properties, in practice some derivatives of the M13KO7 helper phage do not differ from each other. For example, if we compare all the derivatives of the helper phage CM13, it gives larger plaques and higher titers (twice as many CFU/ml), so it is easier to prepare. The CM13 helper phage gives consistently good yields, even at low multiplicity of infection, but has a lower packing factor. At the same time, the M13KO7 helper phage gives similar phage yields, requires precise adherence to bacterial culture density (about 0.5 OD600) for better cell contamination, and ensures a high packing factor. The M13KO7 helper phage is recommended for producing single-stranded DNA, e. g. for Kunkel mutagenesis, due to its better packing ratio. The helper phage R408 is an f1 derivative, does not carry antibiotic resistance and may improve packaging of single-stranded DNA (Dotto et al., 1981).

Hyperphages are a very attractive replacement for conventional helper phages (Rondot et al., 2001). Hyperphages contain a genome with a deletion of the pIII gene. To obtain them, packaging cells from E. coli are used to synthesize functional pIII, which is necessary for the formation of a complete particle and packaging of the phage genome. The resulting hyperphages carry functional pIII on their surface, but their genome is defective in the pIII gene. Each of the resulting phages carries multiple copies of the antibody or peptide on its surface, which greatly increases panning efficiency.

**Fig. 6.**
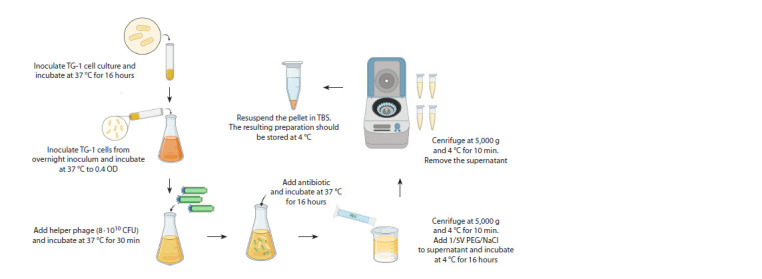
Scheme of electroporation

Although the helper phage is commercially available, for convenience it is useful to amplify a helper phage that is as good as the commercial phage in terms of biological titer. One of the important criteria for helper phage amplification is the optical density of the infected E. coli cell culture; the optimum value is usually 0.4 OD (Fig. 6). Higher densities result in a large percentage of non-transduced bacteria not producing virions, while lower densities can increase mismatches caused by differences in phage growth rate and lower phage production, as well as the expression of toxic fusion proteins that limit bacterial growth. The infectious dose of the helper phage is also critical. The optimal dose is 8·1010 CFU/ml; the excess usually leads to overconsumption of the preparation.

When infecting cells with a helper phage, incubation should be performed without agitation. Agitation promotes cell growth; therefore, not all the cells will be infected with a helper phage. It is also important to respect the time intervals: incubation of the cell culture with a helper phage at +37 °C should not exceed 30 minutes. Extraordinary growth of helper phage-infected cells is achieved by adding an antibiotic (depending on the antibiotic embedded in the helper phage genome), in most cases kanamycin.

If the helper phage is precipitated with PEG/NaCl solution, the preparation should be incubated for 16 hours at +4 °C. This promotes better precipitation of the helper phage. Precipitation should be performed twice, since some preparation will remain in the supernatant after the first precipitation. The resulting preparation should be dissolved in TBS buffer. The amount of TBS buffer used is chosen individually and depends on the precipitate size. The resulting preparation must be stored at +4 °C.

## Conclusion

In this article, we have attempted to reflect on the main problems faced by researchers dealing with phage library construction and the ways of solving them. In our opinion, one of the key problems in the construction of phage antibody libraries is the preparation of the phagemid vector for the insertion of antibody gene diversity, on which the library diversity depends critically. Equally important is amplification and obtaining a purified helper phage preparation, as well as obtaining highly competent cells for electroporation

Despite these methodological challenges, the phage display technique continues to be successfully developed. This technology is attractive for a number of reasons, including its relative simplicity, the ability to screen large numbers of samples in a short time, and the ability to select antibodies specific to a wide range of antigens. Phage display has opened up new prospects for antibody discovery and development. Application of phage libraries of human antibody fragments makes it possible to avoid the use of chimeric or humanized antibodies for therapy, thus avoiding problems associated with the immunogenicity of drugs.

We would like to point out that this article is not an ideal guide but may help one solve some problems in obtaining a phage library of antibodies with a large repertoire

## Conflict of interest

The authors declare no conflict of interest.
